# Brain Abscess due to *Streptococcus intermedius* after Spontaneous Esophageal Perforation in an Adolescent

**DOI:** 10.1155/2024/5593403

**Published:** 2024-05-09

**Authors:** Sandra Mabel Camacho-Gomez, Javier Monagas, Robert Adam Noel, Luis Castagnini

**Affiliations:** ^1^Department of Pediatrics, The University of Texas at Austin, Section of Gastroenterology, Hepatology and Nutrition, Dell Children's Medical Center, Austin, Texas, USA; ^2^Department of Pediatrics, Baylor College of Medicine, Section of Gastroenterology, Hepatology and Nutrition, The Children's Hospital of San Antonio, San Antonio, Texas, USA; ^3^Vaccine Clinical Research, Merck Research Laboratories, North Wales, Pennsylvania, USA

## Abstract

*Streptococcus intermedius* is an inhabitant of the oral cavity and gastrointestinal tract, known to cause deep-seated abscesses. Thereby, we present a previously healthy adolescent with esophageal perforation (EP) and secondary mediastinal and brain abscesses due to *Streptococcus intermedius*. EP is a potentially life-threatening condition that requires a prompt diagnosis.

## 1. Introduction

*Streptococcus intermedius* is a Gram-positive bacteria found in the oral cavity and gastrointestinal and respiratory tract, known to cause abscesses [1]. Scattered reports of *Streptococcus intermedius* invasive infections have been described in the pediatric literature, and different mechanisms of disease invasion are postulated. Abscess formation is secondary to invasion of the sterile site by dissemination or contiguous infection after iatrogenic or accidental inoculation [2–6]. We report the clinical presentation and management of an immunocompetent pediatric patient with cough, fever, and altered mental status diagnosed with esophageal perforation (EP) and mediastinal and brain abscesses due to *Streptococcus intermedius*. The EP was treated with conservative management and endoscopic approach by creating a hood with endoclips. This case highlights the diagnosis and management of EP and brain abscesses caused by *Streptococcus intermedius*.

## 2. Case Presentation

A previously healthy, 15-year-old white male presented to the emergency department with 3-week history of a persistent productive cough followed by one week of fever up to 103°F (39.4°C), headache, and progressive altered mental status for 24 hours. Two weeks earlier, he was prescribed azithromycin for a suspected atypical pneumonia, for which he only took one dose. Additional symptoms, including chest pain, were denied. The past medical history was relevant for occasional episodes of epistaxis and recurrent otitis media. He denied being sexually active or taking any recent medications other than azithromycin. On physical exam, he was afebrile, somnolent, but oriented to place, time, and person, with a Glasgow coma scale of 15. The remainder of his examination, including auscultation of all lung fields, cardiac function, and evaluation of the tympanic membranes, was normal.

Initial laboratory evaluation included an elevated C-reactive protein (CRP) level of 20.54 mg/dL (195.2 mMol/L, normal values <0.5 mg/dL), a white blood cell count of 21,900/*μ*L (21.9 × 10^9^/L) with 85% neutrophils and 9% lymphocytes, hemoglobin of 11.6 g/dL (116 g/L), and a platelet count of 367 × 10^3^/*μ*L (367 × 10^9^/L). He was admitted to the pediatric inpatient unit with the concern of a central nervous system infection, and further laboratory and radiology investigations revealed the diagnosis.

**Figure 1:**
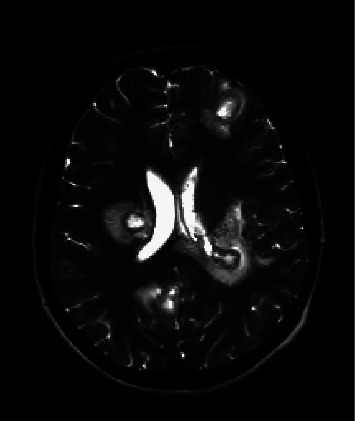
Brain magnetic resonance imaging demonstrating ring-enhancing lesions throughout the brain parenchyma and cerebellum consistent with abscesses on admission.

A head computed tomography (CT) demonstrated brain lesions on admission, and further investigation to better delineate the lesion was obtained with brain magnetic resonance imaging (MRI). The MRI revealed ring-enhancing lesions throughout the brain parenchyma and cerebellum which were consistent with abscesses (Figure 1). Following a lumbar puncture for cerebrospinal fluid (CSF) analysis, he was started on vancomycin, ceftriaxone, acyclovir, metronidazole, and anti-tuberculosis medication due to the initial and protracted respiratory symptoms for 3 weeks, suggesting an infection that started as a respiratory illness. The presence of cough and later fever and the MRI findings raised the concern for central nervous system tuberculosis. He had 3 weeks of insomnia and agitation. He was started on levetiracetam as prophylactic therapy for seizures after a normal electroencephalogram.

**Figure 2:**
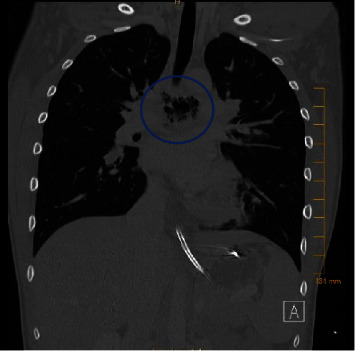
Chest computed tomography revealing a mediastinal abscess.

**Figure 3:**
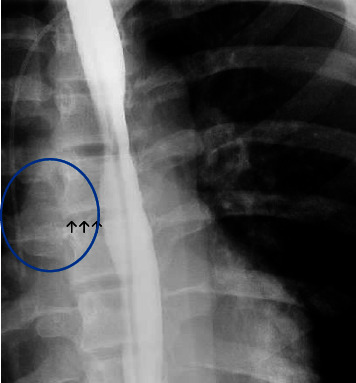
Esophagram showing contrast leakage due to esophageal perforation on the initial presentation. Arrows indicate the fistulous tract location, and the blue circle shows the mediastinal abscess area.

A chest CT (Figure 2) showed a mediastinal abscess associated with a possible EP, which was subsequently confirmed by an esophagram. The esophagram revealed leakage of contrast through a 1.3 mm tract (Figure 3). To obtain a culture from the source of infection, the patient underwent esophagogastroduodenoscopy with ultrasound guidance, which showed a nodule with a superficial ulceration in the mid-esophagus concerning for a fistula. The endoscopic ultrasound revealed an organized wall with a heterogeneous center and cystic spaces, consistent with an abscess. The fistulous tract was cannulated and the cultures sent from the fluid were negative.

The diagnostic workup was negative for *Mycobacterium tuberculosis*, toxoplasmosis, neurocysticercosis, HIV, histoplasmosis, cryptococcosis, blastomycosis, and free-living amoebas. Complete diagnostic workup results including extensive investigation for infectious etiologies can be found in Supplementary Table 1 (laboratory results and infectious disease evaluation). CSF sample for 16rRNA PCR returned positive for *Streptococcus intermedius* confirming the diagnosis of bacterial brain abscesses.

**Figure 4:**
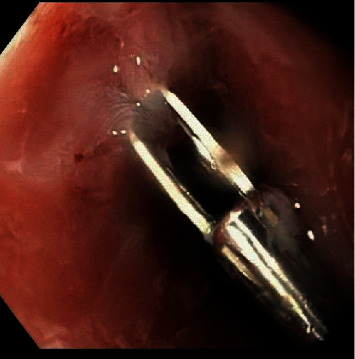
Endoclip placed above the fistulous tract to create a hood via esophagoscopy and protect the abscess cavity from further contamination with feeding, on week 6 of hospitalization.

A bronchoalveolar lavage sample was negative for acid-fast bacilli as well as negative for aerobic and anaerobic organisms. Anti-tuberculosis therapy was discontinued. He was managed conservatively with antibiotics and nasogastric feeding tube for 6 weeks. Due to persistent fistula on esophagram, an endoscopy was performed to place endoclips above the fistulous tract to create a mucosal hood to protect the abscess cavity from further contamination with oral feeding (Figure 4). At this point, he was able to start on a soft mechanical diet. Follow-up esophagram at 8 weeks revealed interval healing of the EP without contrast leakage. The white blood cell count and CRP levels normalized, and the brain MRI showed improvement of the lesions.

He was discharged with a peripherally inserted central catheter to complete a total of 8 weeks of antibiotic therapy with ceftriaxone, after which he was transitioned to oral amoxicillin with clavulanic acid to complete a 10-week total course. On follow-up, he was asymptomatic and almost back to baseline activity. After a normal modified barium swallow study, he was started on a regular diet.

## 3. Discussion

This patient developed bacterial brain abscesses as a complication from an EP. His initial presentation consistent with pneumonia with a violent cough and/or mucosal defect may be responsible for the development of EP and subsequent mediastinal abscess due to *Streptococcus intermedius.* The mediastinal abscess subsequently progressed into brain abscesses due to transient bacteremia. This case highlights two important learning points: (1) diagnosis and management of brain abscesses caused by *Streptococcus intermedius* and (2) diagnosis and management of EP.

*Streptococcus intermedius* is a Gram-positivebacterium member of the *Streptococcus anginosus* group, also known as the *Streptococcus milleri* group [1]. These organisms are normally found in the oral cavity and gastrointestinal and genitourinary tracts. Invasive disease occurs when the organisms invade sterile sites and form abscesses [2]. *Streptococcus intermedius* virulence factors include hyaluronidase and neuraminidase [3]. These enzymes enhance the spread and extension of infection [3] by destroying the extracellular matrix of connective tissues [4].

Disseminated infections are commonly associated with dental procedures [5], trauma, and congenital heart disease [6]. Localized infections are a consequence of contiguous extension from the respiratory tract in cases of otitis, sinusitis [6], and pneumonia [7], which correlates with our patient presented with respiratory tract infection complicated with contiguous mediastinal abscess.

Serious *Streptococcus intermedius* infections also include abscesses of brain, lymph nodes, liver, pelvis, and lung [1]. The multifocal abscesses are often secondary to seeding from a distant source after bacteremia, as shown in this case [8]. We concluded that the 15-year-old with mediastinal abscess had hematogenous dissemination to the brain. Though he recovered almost to baseline, in children, brain abscesses are associated with permanent neurologic impairment and death [9].

Antimicrobial therapy should be tailored to the presumptive microorganisms and microbiological results [8]. *Streptococcus intermedius* is generally sensitive to penicillin, cephalosporins, vancomycin, linezolid, and daptomycin [1]. Adequate anti-infective therapy of the brain abscess was demonstrated by clinical improvement and a gradual decrease in the size; total duration of the treatment should be for a minimum of 4 to 8 weeks [8].

Surgical management is often the next step for patients who fail medical management or for abscesses that are located in the posterior fossa, multiloculated, or superficially circumscribed abscesses [8]. Surgery was not warranted in our case given his favorable clinical and radiologic response to antibiotics.

A posttraumatic esophageal tear has been reported in an adult patient after a truck accident and subsequent abscess culture was found positive for *Streptococcus intermedius* [10] but members of the *Streptococcus anginosus* group are difficult to grow with conventional methods [6]. This likely explains sterile cultures in our patient. Newer molecular techniques, like PCR and electrospray ionization with mass spectrometry (PCR/ESI-MS), have been demonstrated to be useful in the microbiological diagnosis of this infection [11]. A prompt diagnosis is important for a favorable prognosis [12].

EP is a potentially life-threatening condition with high morbidity and mortality [13]. The most common location for EP in children is in the thoracic portion [14], and the most common cause is iatrogenic [15]. Chest pain is the cardinal symptom of EP, usually acute and sudden onset, with radiation to the back or left shoulder [13]. Other signs and symptoms include tachycardia, tachypnea, fever, and leukocytosis [14]. Although the patient did not report chest pain or other cardiac symptoms, he exhibited neurological changes consistent with meningitis and brain abscesses, due to dissemination from the mediastinal abscess.

Spontaneous EP, known as Boerhaave syndrome, can present as a result of retching and vomiting, usually at the left posterolateral area above the cardia of the stomach, and is due to the weakness of the muscular layer and lack of connective tissue support [16].

Systemic inflammatory markers rise following a perforation [13], although the elevated CRP in our patient might be secondary to the systemic infection. Chest X-ray typically shows pleural effusion, pneumomediastinum, subcutaneous emphysema, and hydrothorax, pneumothorax, or pulmonary collapse [13]. If the X-ray results are negative, repeating the study is necessary if EP is highly suspected, and a flexible endoscopy should be considered [13]. The use of barium soluble contrast should be avoided due to the high risk of mediastinitis, but the use of water-soluble contrast is helpful in identifying esophageal leaks [13]. A CT image of the chest can give information regarding the location and the degree of containment [13].

Nonoperative approach with endoscopy management should be considered when the clinical situation of the patient allows [13]. Surgical intervention should be considered depending on the severity of the EP [17]. Factors such as location, size, age of the patient, the duration of the perforation, inflammatory markers (leukocyte count, CRP, and signs of sepsis), etiology, nutritional status, and underlying medical conditions play an important role in this decision [17].

The management of EP includes a prompt diagnosis, close monitoring of the clinical status of the patient, antibiotic therapy, nutritional support, and control of contamination [18].

## 4. Conclusions

The patient initially had pneumonia with a violent cough and subsequently developed a mediastinal abscess due to *Streptococcus intermedius*. He then could have had a transient bacteremia with septic emboli into the brain parenchyma causing brain abscesses. The patient was promptly initiated on broad-spectrum antibiotic therapy, and the mediastinal abscess was drained with the help of endoscopic ultrasound guidance. The EP was successfully managed with conservative management and an endoscopic approach.

This case is based on a presentation by Drs. Camacho-Gomez, Monagas, and Noel at the Annual Meeting of the North American Society for Pediatric Gastroenterology and Nutrition 2019, Chicago, Illinois, Poster Session I, October 17, 2019, Poster Number 175 [19].

## Data Availability

The background data used to support the findings of this case report are included within the article.

## Abbreviations

EP: Esophageal perforation
CRP: C-reactive protein
CT: Computed tomography
MRI: Magnetic resonance imaging
CSF: Cerebral spinal fluid.

## Supplementary Materials

Supplementary Materials The complete diagnostic workup results including extensive investigation for infectious etiology can be found in the supplemental Table 1 and the timeline of clinical and diagnostic events can be found in Table 2.
