# Supplementary material for: Brain Abscess due to Streptococcus intermedius after Spontaneous Esophageal Perforation in an Adolescent
Source: Case Rep Pediatr. 2024 May 9;2024:5593403. doi: 10.1155/2024/5593403 (PMC11098600; doi:10.1155/2024/5593403)
Supplement: Supplementary Materials — The complete diagnostic workup results including extensive investigation for infectious etiology can be found in the supplemental Table 1 and the timeline of clinical and diagnostic events can be found in Table 2. [file 5593403.f1.zip › supplemental 2.pdf]

Admission

-Brain MRI: showed cerebellum/brain lesions. (Figure A).

-Chest CT: demonstrated mediastinal abscess and possible EP (Figure B).

-Esophagram with a leakage of contrast concerned for EP (Figure C).

-Started on Vancomycin, ceftriaxone, acyclovir and Metronidazole after lumbar puncture.

- EGD with Ultrasound (2 days after admission) revealed an organized wall and a heterogeneous center with cystic spaces.

6 weeks

-Esophagram leakage due to persistent fistula.

-EGD with endoclip to create a mucosal hood (Figure D).

-Positive universal 16S rRNA PCR for *Streptococcus Intermedius* on Cerebrospinal fluid.

-Managed with a nasogastric feeding tube for 6 weeks.

8 weeks

-Esophagram: showed an interval healing of the EP without contrast leakage.

-Brain MRI: demonstrated interval improvement of his brain lesions.

-Completed IV metronidazole and ceftriaxone for 8 weeks.

-Transitioned to a soft mechanical diet by mouth.

10 weeks

-Almost recovered to his normal baseline activity on follow-up.

-Completed oral amoxicillin with clavulanic acid for 2 weeks.

-Regular diet

Supplemental Table 2: Timeline of significant clinical and diagnostics events.
MRI (magnetic resonance imaging), CT (computed tomography), EP (esophageal perforation), EGD (esophagogastroduodenoscopy).
