# Supplementary material for: Brain Abscess due to Streptococcus intermedius after Spontaneous Esophageal Perforation in an Adolescent
Source: Case Rep Pediatr. 2024 May 9;2024:5593403. doi: 10.1155/2024/5593403 (PMC11098600; doi:10.1155/2024/5593403)
Supplement: Supplementary Materials — The complete diagnostic workup results including extensive investigation for infectious etiology can be found in the supplemental Table 1 and the timeline of clinical and diagnostic events can be found in Table 2. [file 5593403.f1.zip › supplemental1reviewnotracks.docx]

Supplemental Table 1: Laboratory results and infectious disease evaluation.

|  |  |
| --- | --- |
| Cerebrospinal Fluid (on admission) |  |
| White blood cell count  (/mm^3^) | 6000 |
| Glucose(mg/dL) | 11 |
| Protein (mg/dL) | >200 |
| India Ink stain | Negative |
| Gram stain | Negative |
| Aerobic culture | Negative |
| Fungal culture | Negative |
| Infectious disease evaluation |  |
| Sputum for Acid-fast bacilli smear and culture | Negative |
| Blood culture | Negative |
| Quantiferon | Negative |
| Mycobacterium tuberculosis Complex PCR | Negative |
| Amoeba Panel | Negative |
| Histoplasmosis Immunodiffusion | Negative |
| Histoplasma antigen serum | Negative |
| Histoplasma antigen urine | Negative |
| Histoplasma mycelial | Negative, yeast 1:32 |
| Coccidioides Immunodiffusion | Negative |
| Blastomycosis Immunodiffusion | Negative |
| Cocci EIA and CF | Negative |
| Galactomannan negative | Negative |
| Toxoplasmosis IgM and IgG | Negative |
| HIV antibody and antigen | Negative |
| Cryptococcus antigen | Negative |
| Naegleria | Negative |
| Acanthamoeba | Negative |
| Balamuthia | Negative |
| Cysticercosis AB IB | Negative |
| HHV-6 serum PCR | Negative |
| HHV-6 CSF PCR | Detected |
| E. histolytica Ab | Negative |
| Complement Fixation | Normal |
| NADPH oxidase activity | Normal |
| Mediastinal fluid aerobic culture | Negative |
| Mediastinal fluid anaerobic culture | Negative |
| Mediastinal fluid fungal culture | Negative |
| Mediastinal fluid AFB culture | Negative |
| Mediastinal Specimens pathology | acute and chronic inflammation with central necrosis |
| Bronchoalveolar lavage culture | Negative |
